# Fibroma of tendon sheath around large joints: clinical characteristics and literature review

**DOI:** 10.1186/s12891-017-1736-5

**Published:** 2017-08-30

**Authors:** Kayo Suzuki, Taketoshi Yasuda, Shun Suzawa, Kenta Watanabe, Masahiko Kanamori, Tomoatsu Kimura

**Affiliations:** 1 Department of Orthopaedic Surgery, University of Toyama, 2630 Sugitani, Toyama, Toyama 930-0194 Japan; 2 Department of Human Science 1, University of Toyama, 2630 Sugitani, Toyama, Toyama 930-0194 Japan

**Keywords:** Fibroma of tendon sheath, Intra-articular, Extra-articular, Large joint

## Abstract

**Background:**

Fibroma of tendon sheath (FTS) is a benign tumor arising from the synovium of the tendon sheath that occurs mostly around small joints such as the fingers, hands, and wrist. However, FTS rarely arises around a large joint (knee, shoulder, elbow, and ankle) with intra-articular or extra-articular involvement. The clinical characteristics of FTS arising around a large joint are unclear. An additional 3 cases of FTS arising around a large joint are presented. Furthermore, the published cases and the present cases are reviewed with respect to their clinical characteristics and imaging and histopathology findings.

**Methods:**

The 43 reported cases including the present 3 patients were summarized, and the patients’ profiles, symptoms, sites and locations in the joint involved by FTS, magnetic resonance imaging (MRI) findings, surgical procedures, clinical courses, and cytogenetic analyses were reviewed.

**Results:**

The average age of 26 cases was 40.9 years (range 13–69 years), and about 60% of the patients were male. About 10% of the patients had a past history of trauma to the knee joint. Of the present 3 cases, one case was extra-articular around the elbow joint, one case was extra-articular around the knee joint, and one case was intra-articular involving the knee joint. The common symptoms were pain (62.5%), swelling or palpable mass (54.2%), and limited range of motion of the involved joint (50%). The most commonly involved joint was the knee, with 32 cases (74.4%), followed by the elbow in 5 cases (11.6%), ankle in 4 (9.3%), and shoulder in 2 (4.7%). The tumor typically exhibited iso to low signal intensity on T1-weighted MRI. T2-weighted images showed various patterns, but mostly low signal intensity relative to muscle. The surgical margin was marginal resection in all cases. There were no recurrences after surgery. On chromosomal analysis, only the present Case 3 showed an abnormality.

**Conclusions:**

A total of 43 FTS cases that occurred around large joints were summarized. The most common site was around the knee joint. In FTS cases around large joints, it is necessary to distinguish between various fibroblastic and/or fibrohistiocytic tumors.

## Background

Fibroma of tendon sheath (FTS) was first described by Geschickter et al. in 1949 [1], and Chung and Enzinger published the largest series of 138 cases, reporting their clinical and pathological features [2]. FTS is defined as a benign fibroblastic nodular neoplasm that arises from the synovium of a tendon sheath [3]. Macroscopically, the lesion is well circumscribed and lobulated. Histopathologically, it is composed of spindle or stellate-shaped cells similar to fibroblasts in a densely collagenous stroma. Cytological atypia is generally not seen. Although most lesions are hypocellular, some lesions occasionally show increased cellularity in the peripheral area, resembling nodular fasciitis [4]. The specific histological finding of FTS is the presence of elongated thin-walled vessels or clefts, so-called slit-like spaces [4].

FTS is more common in males than females, with a ratio of 3 to 1, and it occurs in patients aged between the 2nd and 5th decades [2]. It is reported to present with a small, painless, slowly growing mass [2–4]. It occurs mainly around small joints, involving the tendons and tendon sheaths of the finger (47.9%), hands (24.8%), and wrist (10.3%) [5]. FTS occurring around large joints, such as the elbows, shoulders, hips, knees, and ankles, is rarely reported 2.8–4.2% [5, 6]. However, in these reports, cases in which the joints involved were not clear were included. To the best of our knowledge, FTSs clearly arising “intra-articular” within a large joint have been reported in only 18 cases, while there have been 3 cases of “extra-articular” FTS around large joints. In 19 cases, there was no description of whether the mass was intra or extra-articular. An additional 3 cases of FTS, one that was extra-articular around the elbow joint, one that was extra-articular around the knee joint, and one that was intra-articular within the knee, are reported. Furthermore, the literature was reviewed, focusing on the clinical characteristics and the imaging and histopathology findings of FTSs arising around large joints.

## Methods

### Case presentation

This report was approved by the Ethics Committee of the Toyama University Hospital (Toyama, Japan), and all 3 patients gave their written informed, consent for this report.

### Case 1

**Fig. 1:**
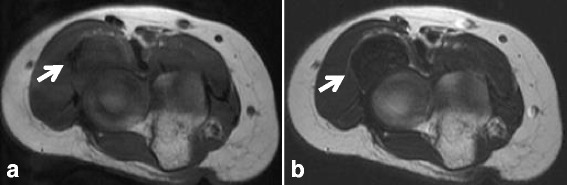
T1- and T2-weighted MR images of Case 1**. a** On T1-weighted images, the mass (arrow) is isointense compared to muscle. **b** On T2-weighted images, the mass (arrow) has low signal intensity compared to muscle

**Fig. 2:**
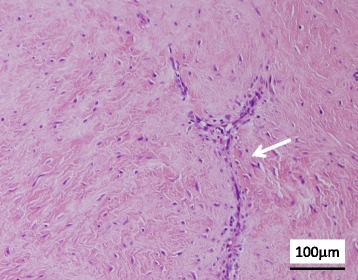
Histopathological findings of the resected tumor. The tumor is composed of spindle cells with low cellularity, and slit-like vessels (*arrow*) are present in the wavy collagenous stroma (hematoxylin and eosin stain)

A 54-year-old woman presented with a 5-month history of a painless mass at the anterior aspect of her right elbow joint. There was no history of trauma. On physical examination, she reported radiating numbness to the right thumb with Tinel’s sign related to the mass. Radiographs of the elbow were normal. Magnetic resonance imaging (MRI) showed a 3.0 cm × 2.1 cm mass with well-defined margins superficial to the distal tendon of the long head of biceps at the anterior aspect of the proximal radial head. The mass was isointense on T1-weighted images and hypointense on T2-weighted images compared to muscle (Fig. 1). Differential diagnoses included schwannoma, chondroma, and tenosynovial giant cell tumor. Intra-operative diagnosis was performed with the anterior approach to the elbow, and a fibrous tumor without malignant cells was diagnosed. Subsequently, marginal resection was performed. The mass was densely adhered to the distal tendon of the biceps extra-articular around the elbow joint. On histopathologic evaluation, the lesion was composed of spindle cells with low cellularity in a background of wavy collagenous bundles. Slit-like vessels were present in the collagenous stroma (Fig. 2). Based on these findings, the lesion was consistent with FTS. The patient showed no evidence of recurrence 14 months after surgery.

### Case 2

**Fig. 3:**
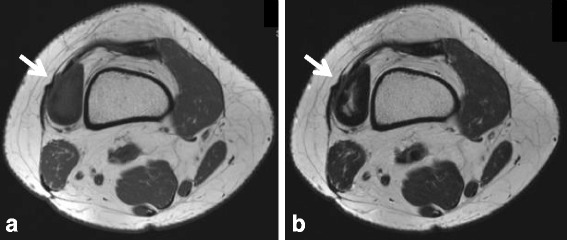
T1- and T2-weighted MR images of Case 2**. a** On T1-weighted images, the mass (arrow) is isointense compared to muscle. **b** On T2-weighted images, the mass (arrow) has high signal intensity in the center of the mass, and the surrounding area has low signal intensity

**Fig. 4:**
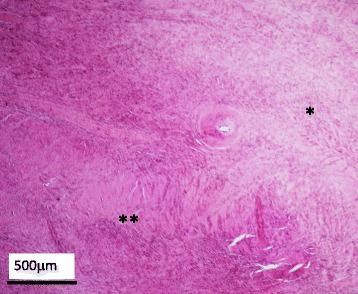
Histopathological findings of the resected tumor. In the low power-field, there are areas where dense proliferation of collagenous tissue is seen (**), and sparsely growing areas (*) and slit-like clefts (*arrow*) are recognized (hematoxylin and eosin stain)

A 42-year-old woman had a 6-month history of a small mass involving the lateral aspect of the knee joint with pain at flexion of the knee joint. There was no history of trauma. Radiographs of the knee were normal. MRI showed a 4.5 cm × 3.0 cm mass with well-defined margins extra-articular around the knee joint, in the depths of the vastus lateralis muscle (Fig. 3). The mass was isointense on T1-weighted images, with a high intensity signal in the center of the mass around low signal intensity on T2-weighted images compared to muscle. Differential diagnoses included a localized type of tenosynovial giant cell tumor. Marginal resection of the mass was performed after needle biopsy. The mass adhered densely to the distal tendon of vastus lateralis between the capsule of the knee joint and the vastus lateralis muscle. On histopathologic evaluation, the lesion was composed of stellate-shaped cells with low cellularity in the background of wavy collagenous bundles and myxoid change in the central zone of the tumor. Slit-like clefts were present in the collagenous stroma (Fig. 4). Based on these findings, the lesion was diagnosed as FTS. The patient presented no evidence of recurrence at 4 months after surgery.

### Case 3

**Fig. 5:**
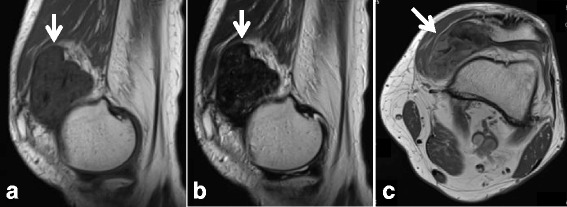
MR images of Case 3**. a** On T1-weighted images, the lesion (arrow) is heterogeneously isointense and hypointense compared to muscle. **b** On T2-weighted images, the lesion (*arrow*) is heterogeneously hypointense. **c** On contrast-enhanced T1-weighted images, the lesion (*arrow*) shows mild and patchy contrast enhancement

**Fig. 6:**
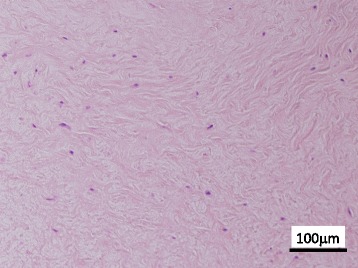
Histopathological findings of the resected tumor**.** The tumor consists of spindle cells resembling fibroblasts with a background of rich collagenous stroma (hematoxylin and eosin stain)

A 63-year-old man had a 4-year history of anterior knee pain on walking and mild swelling of the knee joint. He had no history of trauma. Physical examination showed limited range of motion of the knee joint from 0 to 100 degrees. The mass was located medial and superior to the patella and showed mild tenderness. Radiographs of the knee were normal. MRI presented a lobulated mass, 6 cm × 3 cm, superior to the patella. Whether the mass was intra-articular or extra-articular to the knee joint was unclear on MRI findings (Fig. 5). The lesion showed heterogeneously isointense and hypointense signals to muscle on T1-weighted images and a heterogeneously hypointense signal on T2-weighted images. On contrast-enhanced T1-weighted images, the mass showed mild and patchy contrast enhancement. The preoperative differential diagnoses included the diffuse type of tenosynovial giant cell tumor. Intra-operative diagnosis was performed with a medial parapatellar approach for the knee, and a fibrous tumor without malignant cells was diagnosed. The patient then underwent marginal excision of the mass. The mass was located in the synovial membrane of the suprapatellar capsule within the knee joint. Gross examination of a cut section of the surgically resected mass revealed a grayish white, well-circumscribed, lobulated lesion with a thin capsule. On histological examination of the lesion, spindle cells resembling fibroblasts with a background of collagenous stroma were seen (Fig. 6). Most lesions showed little cytological atypia, and cell density was hypocellular. Slit-like clefts were seen. FTS arising from the capsule of the suprapatellar pouch was diagnosed. The patient showed full range of motion without pain and no evidence of recurrence 3 months after surgery.

### Collection of reports

A PubMed search was performed to identify cases of FTS around large joints. The search term was “fibroma of tendon sheath AND joint OR intraarticular”. Forty papers were identified, and 23 papers were reported in detail. The following items were investigated in 43 cases (40 cases described in 23 papers [2, 6–27] and the present 3 cases).

### Patients’ profile

Age, sex, and past trauma history were examined in cases of FTS arising around large joints.

### Symptoms

The types of subjective symptoms, tumor size, and the time to initial consultation were also investigated.

### Site and location

The involved joint and the site and locations in the joint were examined. The site was classified as intra or extra-articular. Based on the MRI findings or intraoperative findings, the locations where the tumors originated were identified.

### Imaging characteristics

To clarify the features on imaging, the findings of radiographs, computed tomography (CT), and MRI were reviewed. Signal intensities on T1 and T2-weighted MRI were examined; signal intensities of FTS were classified as high, iso, and low compared to muscle.

### Surgery and clinical course

The surgical procedures and clinical course, focusing particularly on local recurrence, were reviewed.

### Cytogenetic analysis

Representative fresh tissue from the surgically resected samples of the present 3 cases underwent conventional cytogenetic analysis. Culturing, harvesting, and preparation of slides were performed as previously described [28]. Briefly, the tissues were disassociated mechanically and enzymatically and cultured at 37 °C in RPMI 1640 (Sigma-Aldrich, St. Louis, MO) supplemented with 20% fetal bovine serum (ICN Biomedicals, Inc., Aurora, OH) for 3–8 days. Cultured cells received overnight exposure to colcemid (0.02 μg/ml). Following hypotonic treatment (0.8% sodium citrate for 20 min at room temperature), the cells were fixed 3× with methanol: glacial acetic acid (3:1) at room temperature. Chromosome analysis was performed on the G-band by trypsin and Giemsa (GTG)-banding. GTG banding was performed by incubating the glass slides in a 0.05% trypsin solution at 37 °C for 15 s, followed by rinsing the slides in phosphate-buffered saline buffer and staining in 5% Giemsa stain for 8 min. The slides were rinsed with water and air dried. The karyotypes were expressed according to the International System for Human Cytogenetic Nomenclature 2013 [29].

## Results

### Patients’ profile

The 43 reported cases, including the present 3 patients, of FTS around large joints, such as elbows, shoulders, knees, and ankles, were reviewed (Table 1) [2, 6–27]. The average age of 26 cases for whom the information was available was 40.9 years (range 13–69 years), and about 60% of the patients were male. Two of 21 cases (9.5%) had a past history of trauma to the knee joint [11, 21].

**Table 1:** The clinical characteristics of fibroma of tendon sheath around large joints

Author (No. of ref.)	NO.	Case	Involved Joint	Site	Location	Symptom	Past history	Duration (M)	Size	Operation
Chung EB [2]	7	ND	knee	ND	ND	ND	ND	ND	ND	ND
	3	ND	elbow	ND	ND	ND	ND	ND	ND	ND
	3	ND	ankle	ND	ND	ND	ND	ND	ND	ND
Hashimoto H [7]	3	ND	knee	ND	ND	ND	ND	ND	ND	ND
	1	ND	elbow	ND	ND	ND	ND	ND	ND	ND
Smith PS [8]	1	33, M	knee	ND	quadriceps tendon	ND	ND	ND	3x2x1	ND
Humphreys S [9]	1	67, M	knee	ND	ND	ND	ND	72	1.5	ND
Ogata K [10]	1	16, F	knee	intra	PCL	effusion	none	4	2 × 1.5 × 1	AS
Pinar H [11]	1	38, M	knee	intra	suprapatellar capsule	swelling	trauma	5	7x4x1.5	AS & open
Hur J [12]	1	13, M	knee	intra	infrapatellar fat pad	mass	ND	9	3.5 × 4.3 × 3.2	ND
McGrory JE [13]	1	38, M	knee	intra	patellar tendon	pain, mass, limited ROM	ND	ND	ND	Open
Hitora T [14]	1	50, M	knee	intra	posterior capsule	mass	none	4	5x4x3	Open
Takakubo Y [15]	1	39, M	knee	intra	PCL & posterior capsule	limited ROM	none	4	3.2x3x2.3	AS
Ahn JH [16]	1	49, M	knee	intra	posterior capsule	effusion, limited ROM	none	24	3.5 × 1.2 × 1	AS
Le Corroller T [17]	1	18, M	knee	extra	semimembranous bursa	pain, swelling	none	ND	2.5	Open
Okada J [18]	1	69, M	knee	intra	infrapatellar fat pad	pain, swelling	none	5	1.5x4x3.5	Open
Aynaci O [19]	1	42, F	knee	intra	infrapatellar fat pad	pain, locking, limited ROM	none	10	2.5 × 1.5 × 0.3	Open
Moretti VM [6]	1	51, M	knee	extra	superficial to MCL	pain, mass	none	2	6 × 4.8 × 2.1	Open
	1	35, F	knee	intra	patellar tendon	pain, mass, limited ROM	none	9	3.4 × 4.2	Open
	1	42, M	knee	intra	patellar tendon	pain, mass, limited ROM	none	6	2.0	Open
Kundangar R [20]	1	35,?	knee	intra	PCL & posterior capsule	pain, mass, limited ROM	none	5	3x2x2	AS
Griesser MJ [21]	1	17, M	knee	intra	PCL & posterior capsule	pain, limited ROM	trauma	1	3.3 × 1.7 × 1.9	Open
Ciatti R [22]	1	58, M	ankle	intra	anteromedial capsule	swelling, limited ROM	none	24	5.5 × 3.4 × 2.6	Open
Hermann G [23]	1	30, F	shoulder	intra	joint space	pain	none	1.5	ND	Open
De Maeseneer M [24]	1	49, F	shoulder	extra	biceps long head	pain, limited ROM	ND	12	5	Open
Ha DH [25]	1	45, M	knee	intra	lateral capsule	pain, ITB friction syndrome	none	ND	ND	AS
Rathore S [26]	1	16, F	knee	intra	ITB	pain, ITB friction syndrome	none	2–3	4.1 × 2.7 × 0.8	Open
Toki S [27]	1	54, M	knee	intra	patellofemoral retinaculum	pain, limited ROM	none	3	4.5 × 2.0 × 5.5	Open
Present Case 1	1	54, F	elbow	extra	distal tendon of biceps	mass, Tinel’s sign	none	5	3 × 2	Open
Case 2	1	42, F	knee	extra	distal attachment of VL	Mass	none	7	4.5 × 3	Open
Case 3	1	63, M	knee	intra	suprapatellar capsule	pain, limited ROM	none	6	6 × 3	Open

*No. of ref*. number of reference, *NO* number of cases, *Duration (M)* duration of symptom (months), *ND* not described, *M* male, *F* female, *PCL* posterior cruciate ligament, *AS* arthroscopy, *ROM* range of motion, *MCL* medial collateral ligament, *ITB* iliotibial band, *VL* vastus lateralis

### Symptoms

Clinical characteristics and symptoms of FTS around large joints, where available in the reported cases, are listed in Table 1. The common symptoms reported were pain, reviewed in 15 (62.5%) of 24 cases [6, 10–27], 13 (54.2%) patients presented with swelling or a palpable mass, and 12 (50%) experienced decreased range of motion at the involved joint. The size of FTSs that occurred around large joints was not large, but relatively small, with no cases exceeding 7 cm.

### Location and site

The most commonly involved joint was the knee, with 32 cases (74.4%), followed by the elbow in 5 cases (11.6%), ankle in 4 (9.3%), and shoulder in 2 (4.7%). In 24 cases, it was clearly reported where the fibroma occurred; 19 cases were intra-articular within a large joint, and 5 were extra-articular. There were 17 intra-articular cases involving the knee joint, mostly arising from the posterior cruciate ligament (PCL) and/or posterior capsule in 6 cases. Extra-articular FTSs around the knee joint were seen in 3 cases, involving the superficial part of the medial collateral ligament (MCL) or attachment of muscle. Based on these reviewed cases, intra-articular FTSs around large joints seem to occur mostly around the knee joint, and they originated in the joint capsule.

### Imaging

The imaging features of FTSs that originated around large joints are shown in Table 2. Radiographs and CT are usually normal, but bone erosion may rarely be seen [15, 21]. MRI is useful to evaluate the local presence and properties of FTSs. T1-weighted images often show a well-defined lesion that is low or isointense to muscle [6, 11–25, 27]. T2-weighted images showed various patterns, most commonly low signal intensity to muscle in 8 of 43 reviewed cases [6, 13, 22, 23, 26, present cases 1 and 3], a central high intensity signal in a low intensity area and a focally high intensity signal in a low area in 5 cases [12, 16, 19, 27, present Case 2], and a high intensity signal was seen in 4 reports [11, 18, 20, 25]. A few cases showed a heterogeneous low or high-intensity mass or mixed high and low signals [6, 15, 17, 21, 24].

**Table 2:** Imaging findings of fibroma of tendon sheath around large joints

Author (No. of ref.)	Case	Bone erosion	MRI		
			T1WI	T2WI	Enhancement
Pinar H [11]	38, M	-	iso	high	rim enhancement
Hur J [12]	13, M	-	low	low and central high	ND
McGrory JE [13]	38, M	-	heterogeneous low	ND	ND
Hitora T [14]	50, M	-	low	low	ND
Takakubo Y [15]	39, M	+	low	high and low	peripheral enhancement
Ahn JH [16]	49, M	-	low	low and focally high	ND
Le Corroller T [17]	18, M	-	low	heterogeneously low	peripheral enhancement
Okada J [18]	69, M	-	low	high (T2*)	peripheral enhancement
Aynaci O [19]	42, F	-	low	low and focally high	ND
Moretti VM [6]	51, M	-	low	heterogeneously low	patchy enhancement
	35, F	-	low	low	ND
	42, M	-	iso	low to iso	ND
Kundangar R [20]	35,?	-	iso	high	rim enhancement
Griesser MJ [21]	17, M	+	iso	heterogeneously high	focally enhancement
Ciatti R [22]	58, M	-	low	low	ND
Hermann G [23]	30, F	-	low	low	ND
De Maeseneer M [24]	49, F	-	iso	high and low band	patchy enhancement
Ha DH [25]	45, M	-	low	high	ND
Rathore S [26]	16, F	-	ND	low	ND
Toki S [27]	54, M	-	iso	low and focally high	slightly enhancement
Present Case 1	54, F	-	low	low	ND
Case 2	42, F	-	low	low and central high	rim enhancement
Case 3	63, M	-	low	low	rim and lobular enhancement

*No. of ref.* number of reference, *MRI* magnetic resonance imaging, *T1WI* T1-weighted imaging, *T2WI* T2-weighted imaging, *M* male, *F* female, *ND* not described

### Surgery and clinical course

Of the 23 cases in which surgical methods were described, 5 patients underwent tumor resection by arthroscopy [10, 15, 16, 20, 25], while 18 underwent open surgery [6, 11, 13, 14, 17–19, 21–24, 26, 27 and the present cases]. Cases with tumors larger than 3 cm tended to be resected by open surgery with marginal margin in all cases. There were no cases of recurrence after surgery.

### Cytogenetic analysis

**Fig. 7:**
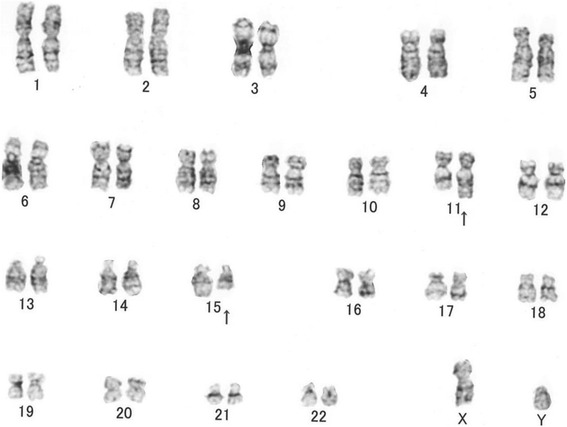
Karyotyoe of intra articular FTS in Case 3**.** A representative karyotype of Case 3 showing a t(11;15)(q23;q22) translocation (*arrows*)

In the cases reviewed, no patients underwent cytogenetic analysis. Among the present cases, chromosomal analysis was performed in Cases 2 and 3. Case 2 showed a normal karyotype: 46,XX [20 cells], while Case 3 exhibited the following abnormal chromosomal complement: 46,XY,del(9)(q?),der(16)add(16)(p11.2)add(16)(q24)[2 cells]/46,XY,t(11;15)(q23;q22)[1 cell]/46,XY,?t(5;14)(q35;q11.2)[1 cell]/46,XY[16 cells] (Fig.7).

## Discussion

### Clinical characteristics and Karyotype

FTS is defined as a benign fibroblastic nodular neoplasm that arises from the synovium of a tendon sheath [3]. FTS almost involves the small joints, such as fingers and wrists, accounting for 80–85% of cases [5]. Particularly, FTSs arising around a large joint is rare condition. Of the FTSs occurring around large joints, the most frequently involved joint was the knee. Among FTSs arising within the knee joint, they often occurred in the PCL and/or posterior capsule. FTSs typically originate from tendons or tendon sheaths [2]. On histological examination, the tendon sheath typically shows layers that are continuous with the outer fibrous tendon sheath and the inner synovial sheath layer with vessels [30]. The histological structure of the joint capsule is similar to the structure of the tendon sheath at the point where the joint capsule consists of two layers, the outer dense fibrous connective tissue and the inner synovial layer with vasculature [31, 32]. Therefore, FTSs might also occur from the joint capsule, not only the tendon or tendon sheath. The knee joint is the most commonly involved joint for FTSs around large joints [2, 6–27]. The present Case 3 originated from the suprapatellar capsule of the knee joint, and the mass was covered with synovial membrane identified by histopathological examination. Therefore, intra-articular FTSs might originate from the synovial membrane of the inner layer of the knee joint capsule.

The common symptoms of FTSs around a large joint reported were pain, reviewed in 15 (62.5%) of 24 cases, 13 (54.2%) patients presented with swelling or a palpable mass, and 12 (50%) experienced decreased range of motion at the involved joint. Although it is rare, a joint effusion and locking appear in a few cases [10, 16, 19]. In 3 extra-articular FTSs arising around a large joint, the symptom was often pain with joint motion [6, 17, 24], and they rarely showed radiating pain due to irritating tissue surrounding the tumor, for example the nerve, as in the present Case 1.

The karyotype of FTS was presented in 2 previous case reports (Table 3) [33, 34]. In both cases, translocation with a break point was observed on the long arm of chromosome 11 [33, 34]. In the present Case 3, only one cell showed translocation involving the long arm of chromosome 11. FTSs may be associated with an abnormality of the long arm of chromosome 11. However, how the rearrangement of the long arm of chromosome 11 was related to tumorigenesis of FTS was not clear.

**Table 3:** Karyotype of fibroma of tendon sheath

Author (No. of ref.)	Age, Sex	Location	Karyotype
Dal Cin P [35]	60, F	thumb	t(2;11)(q31–32;q12)[10]/46,XX[10]
Nishio J [36]	38, F	hand	46,XX,t(9;11)(p24;q13–14)[10]/46,XX,t(1;20)(p13;q13.1)[2]/46,XX,t(1;2)(q21;q35)[1]/46,XX,t(3;16)(p21;q24)[1]/46,XX[3]
Our Case 2	42, F	knee	46,XX[20]
Our Case 3	63, M	knee	46,XY,del(9)(q?),der(16)add(16)(p11.2)add(16)(q24)[2]/46,XY,t(11;15)(q23;q22)[1]/46,XY,?t(5;14)(q35;q11.2)[1]/46,XY[16].

*No. of ref*. number of reference, *M* male, *F* female

### Imaging

Radiographs and CT are usually normal. MRI is useful for diagnosis of tumor localization and properties. FTSs typically show iso to low signal intensity on T1-weighted images [35]. However, on T2-weighted images, FTSs shows various signal changes. Generally, tumors with low signal intensity on T2-weighted images have significant fibrous elements and marked hypocellularity [36]. Therefore, because the FTSs almost showed hypocellular mass, the signal intensity of FTSs on T2-weighted images was mostly low [6, 14, 22, 23], including the present cases. But the high signal intensity was mixed in hypointense areas in some FTSs [12, 15, 16, 19]. Fox et al. reported that the MRI findings reflected well the histological features in FTS [35]. Occasionally, on T2-weigted imaging, the tumor showed high signal intensity centrally and slightly low signal peripherally. These findings presented that the tumor contained central areas of increased cellularity and myxoid change on a background of slit-like vascular [35].Various types of gadolinium-enhanced MRI findings are seen from case to case. The patterns of enhancement are reported as rim/peripheral [11, 15, 17, 18], patchy or focal in the mass [6, 21, 24], and lobular enhancement (present Case 3). Takakubo et al. [15] described peripheral enhancement that may reflect blood vessel proliferation at the periphery of the tumor. Pinar et al. reported that rim enhancement for an intra-articular FTS reflects the existence of synovium around the mass [11].

### Differential diagnosis

The differential diagnostic considerations for FTS occurring around large joints mainly include the giant cell tumor of tendon sheath (GCTTS, aka. Nodular tenosynovitis) [5, 25, 35, 37] and nodular fasciitis [2, 4, 5, 7, 38]. FTS and GCTTS arise in similar location, as finger, wrist, and knee joint. MRI findings of GCTTS typically show a solid soft tissue mass with a round, oval, or multilobular shape near or inside the joint and present low signal intensity on both T1- and T2-weighted images, similar to FTS [37]. Contrast enhancement on MRI is variable for both FTS and GCTTS [24]. Although contrast enhancement is usually more prominent in GCTTS, FTS show lack of contrast enhancement or mild enhancement [24]. Histopathological features of GCTTS are characterized by the existence of multinucleated giant cells and hemosiderin deposits, as this point is important for differential diagnosis [5]. Nodular fasciitis typically originates from the surface of fascia and extends into subcutaneous tissue or occasionally muscle; therefore, intra-articular nodular fasciitis is rare [38]. Clinically, nodular fasciitis presents as a rapidly growing, painful mass. The MRI findings of nodular fasciitis reported by Coyle et al. were predominantly low T1 and heterogeneously increased T2 signal intensities in most of the lesions evaluated, with variable amounts of perilesional reticulated soft tissue edema seen on fluid-sensitive imaging acquisitions in 20 of 29 cases [39].

Nodular fasciitis is thought to histologically resemble FTS. The point of differential diagnosis is that the characteristic vascular pattern of FTS is not seen in nodular fasciitis, and the presence of a less orderly, tissue culture-like growth pattern, more prominent myxoid stroma, and foci of extravasated red blood cells favors the diagnosis of nodular fasciitis [38].

## Conclusion

In conclusion, FTSs arising around large joints (knees, shoulders, elbows, and ankles) are rare. This is the first summary of the 43 reported cases, including the present 3 patients, of FTSs that occurred around large joints. The most commonly involved site was the knee, followed by the elbow. The characteristic symptom of FTS arising around a large joint is pain with joint motion and a limited range of joint motion. The MRI findings are typically iso to low intensity on T1-weighted images and various signal changes on T2-weighted images. Therefore, histopathological analyses are needed for accurate diagnosis. Marginal resection is enough for the treatment of FTS around a large joint; there have been no recurrences in the present 3 cases and the reviewed cases so far.

## Abbreviations

aka: Also known as
CT: Computed tomography
FTS: Fibroma of tendon sheath
GCTTS: Giant cell tumor of tendon sheath
GTG: G-band by trypsin and Giemsa
MCL: Medial collateral ligament
MRI: Magnetic resonance imaging
PCL: Posterior cruciate ligament
PVNS: Pigmented villonodular synovitis
